# Altered Ratio of IFN-γ/IL-10 in Patients with Drug Resistant *Mycobacterium tuberculosis* and HIV- Tuberculosis Immune Reconstitution Inflammatory Syndrome

**DOI:** 10.1371/journal.pone.0046481

**Published:** 2012-10-10

**Authors:** Keira H. Skolimowska, Molebogeng X. Rangaka, Graeme Meintjes, Dominique J. Pepper, Ronnett Seldon, Kerryn Matthews, Robert J. Wilkinson, Katalin A. Wilkinson

**Affiliations:** 1 Clinical Infectious Diseases Research Initiative, Institute of Infectious Diseases and Molecular Medicine, University of Cape Town, Cape Town, South Africa; 2 MRC National Institute for Medical Research, London, United Kingdom; 3 Division of Medicine, Imperial College London, London, United Kingdom; 4 Infectious Diseases Unit, GF Jooste Hospital, Cape Town, South Africa; National Institute of Infectious Diseases, Japan

## Abstract

We have described a clinical relationship between HIV-Tuberculosis Immune Reconstitution Inflammatory Syndrome (TB-IRIS) and anti-tubercular drug resistance. Here we studied the immune response of TB-IRIS patients from whom a drug-resistant (n = 11) or drug-susceptible (n = 25) *Mycobacterium tuberculosis* (MTB) strain was isolated after presenting with TB-IRIS. ELISpot analysis and multiplex cytokine analysis of the supernatant collected from peripheral blood mononuclear cells stimulated overnight with the heat-killed H37Rv MTB laboratory strain was used. Although there was no statistical difference in IFN-gamma ELISpot responses between the two groups, the results point towards higher bacterial load in the drug-resistant patients, possibly due to failed therapy. The ratio between secreted IFN-gamma/IL-10 and IL-2/IL-10 was significantly lower in TB-IRIS patients in whom the cause of TB was a drug-resistant strain compared to those with a fully sensitive strain (p = 0.02). Since host immune responses are dependent on the bacterial load, we hypothesise that the impaired cytokine balance is likely to be caused by the poorly controlled bacterial growth in these patients.

## Introduction

Antiretroviral therapy (ART) reduces the risk of tuberculosis (TB) in HIV-1 infected persons by as much as 80% [1], [2]. The rollout of ART in South Africa has been associated with reduced TB case notification rates in some communities [3]. However, TB remains the most significant clinical challenge to the successful rollout of ART, with a high prevalence of diagnosed and undiagnosed TB in HIV-1 infected persons starting ART [4], that can be further complicated by the immune reconstitution inflammatory syndrome (TB-IRIS) [5]. Two forms of TB-IRIS are recognized: (1) paradoxical, in patients established on TB treatment before ART, who manifest with recurrent or new symptoms and clinical features of TB after initiation of ART; and (2) unmasking TB-IRIS, defined in patients not on treatment for TB when ART is started, but who present with active TB within 3 months of starting ART, with heightened intensity of clinical manifestations and marked inflammatory component to the presentation [6]–[9].

Multidrug resistant (tuberculosis resistant to at least isoniazid and rifampicin), and extensively drug resistant (resistant to isoniazid, rifampicin, any fluoroquinolone, and at least one of three injectable second-line drugs) TB is an increasing concern globally, and the importance in high HIV-1 prevalence settings has been highlighted [10], [11]. Of the nearly 500000 new cases of multidrug-resistant tuberculosis yearly, only 30000 are detected and reported; misdiagnosis causes death, nosocomial and community transmission, and amplification of drug resistance [12], [13]. In South Africa, there are an estimated 13000 cases of MDR-TB emerging each year, however, nationwide estimates mask regional variability. Thus, in Khayelitsha, a densely populated peri-urban township outside Cape Town, where close to 6000 tuberculosis cases were notified in 2008 (giving an estimated case notification rate of 1158/100000/year), the burden of MDR-TB cases was estimated to 51/100000/year, or 4.4% of the cases notified in that year [14].

We have recently described a relationship between TB-IRIS and antitubercular drug resistance in a cohort of 100 patients who were considered likely cases of paradoxical TB-IRIS [9]. The prevalence of unsuspected drug-resistant TB amongst TB-IRIS patients was 10.1%, after exclusion of known rifampicin resistance and alternative opportunistic diseases. The symptoms and signs of TB-IRIS in the context of drug-resistant and drug-sensitive disease were clinically indistinguishable. It has been reported that patients with MDR-TB display impaired Th1 type responses and enhanced regulatory T cell responses compared to patients with drug-susceptible TB [15]. We found that TB-IRIS is associated with hypercytokinemia [16], and therefore hypothesised that there might be a difference in the immune response detectable between TB-IRIS patients with drug-resistant compared to drug-sensitive TB. The association between TB-IRIS and heightened type 1 helper T cell responses to a number of *Mycobacterium tuberculosis* (MTB) antigens, as well as elevated secretion of various cytokines has been described [16] [17]. Here we studied the IFN-gamma ELISpot response to a range of MTB antigens, and 17 cytokines secreted into the supernatant of peripheral blood mononuclear cells stimulated overnight with the heat killed laboratory strain H37Rv of MTB. We describe an altered balance between the Th1 and regulatory responses with decreased Th1 (IFN-gamma and IL-2) and increased IL-10 cytokine secretion in TB-IRIS patients in whom the cause of TB was a drug resistant strain compared to those with a sensitive strain.

## Study Population and Methods

### Patients

The study site and study population has previously been described [9]. This prospective observational study was conducted at GF Jooste Hospital (Cape Town, South Africa), with the catchment area including Khayelitsha. The study was approved by the University of Cape Town Faculty of Health Sciences Human Research Ethics Committee (FHS HREC 337/2004). The study involved 250 consecutively enrolled patients, of whom 100 were described previously [9]. All participants provided written informed consent. Samples for immunological analysis were available from 8 TB-IRIS patients with multi-drug (MDR) resistant and 3 TB-IRIS patients with rifampicin mono-resistant (RM) TB. Cases with fully sensitive (FS) TB, who had samples available for immunological analysis (n = 25), were included as controls.

### ELISpot analysis

Peripheral blood mononuclear cells (PBMC) were separated and set up in the IFN-gamma ELISpot assay as described [17]. Antigenic stimuli were endotoxin free and included the Region of Difference-1 (RD1) encoded ESAT-6 (Rv3875) at a final concentration of 10 µg/ml. Additional antigens included alpha-crystallins Acr1 (encoded by Rv2031c) and Acr2 (encoded by Rv0251c), as well as the MTB cell wall associated 38 kDa protein (encoded by Rv0934), all at 10 µg/ml. Purified Protein Derivative (PPD, SSI Denmark) was used at 5 µg/ml. The heat killed (hk) laboratory strain of MTB H37Rv was used to stimulate cells at a multiplicity of infection of 1 bacillus: 1 cell (MOI = 1∶1, 200000/well). No antigenic stimulus was used as negative control, while the positive control was phytohemagglutinin (PHA, Sigma-Aldrich, 10 µg/ml). Results are expressed as IFN-gamma spot forming cells per million (SFC/10^6^) PBMC.

### Multiplex cytokine analysis

Additional PBMC cultures were set up with MTB hkH37Rv (MOI = 1∶1) as described for 24 hours, and the supernatants from stimulated and unstimulated cultures were collected and cryopreserved for future batched cytokine analysis [16]. These supernatants were available from 7 MDR, 3 RM and 10 FS cases. Measurement of the pro-inflammatory cytokines TNF, IL-1beta, IL-6; anti-inflammatory IL-10; Th1-type IFN-gamma, IP-10 (CXCL10), IL-12p40, IL-2; Chemokines MIP-1alpha (CCL3), MIP-1beta (CCL4), RANTES (CCL5), IL-8 (CXCL8); Th-2 type IL-4, IL-5, IL-13; and growth factors GM-CSF and IL-15, was performed in 96-well filter plates, on the Bio-Plex platform (Bio-Rad Laboratories, Hercules, USA), using customized MilliplexTM kits (MPXHCYTO-60K, Millipore, St Charles, Missouri, USA), following the manufacturer's instructions.

### Statistical analysis

Data were analysed using Graphpad Prism 5 Software (Graphpad Software, Inc.). The normality of samples was assessed using D'Agostino and Pearson omnibus normality test. Normally distributed paired samples were compared by using a student's paired t-test, while non-parametric paired samples were analysed using the Wilcoxon-matched pairs test. Unpaired, non-parametric samples were compared using the Mann-Whitney U test and unpaired, normally distributed samples were compared using the unpaired student's t-test. A p-value<0.05 was considered statistically significant. Data are quoted as median (with interquartile range, IQR) unless otherwise stated.

## Results

The baseline characteristics of the patients are summarised in Table 1. Patients were recruited at the time of paradoxical TB-IRIS diagnosis, and were all on first line therapy for tuberculosis at the time of sampling. There was no statistically significant difference in age, gender, and nadir CD4 count between the patients who had TB-IRIS caused by drug resistant (MDR or RM) or fully drug sensitive (FS) MTB isolates. There was a trend towards higher proportions of patients with previous TB, as well as longer duration of IRIS symptoms in the group with drug resistant isolates, although neither of these differences were statistically significant. C-reactive protein (CRP) was elevated in both groups: median 151 mg/L (IQR 65-208) in the MDR+RM group, and 124 mg/L (IQR 80-180) in the FS group (p = 0.65).

**Table 1:** Baseline characteristics of the patients.

	TB-IRIS with drug resistant MTB isolate (MDR^1^ or RM^2^)	TB-IRIS with fully sensitive (FS) MTB isolate	p value
Number of cases	11	25	N/A
Median age (years, IQR)	28 (26–34)	30 (24–36)	0.93
Gender (female/male)	7F, 4M	14F, 11M	0.73*
Nadir CD4 count (cells/µl, IQR)	50 (17–94)	55 (33–93)	0.69
Previous TB (%, n)	45.5% (5)	32% (8)	0.47*
Duration of TB treatment before ART (median days, IQR)	54 (33–84)	49 (24–84)	0.74
Median days to IRIS (IQR)	14 (8–44)	10 (6–16)	0.11
Duration of IRIS (median days, IQR)	170 (65–229)	101 (48–155)	0.31
C Reactive Protein (CRP, mg/L, median and IQR)	151 (65–208)	124 (80–180)	0.65

1 - multi-drug resistant;

2 - rifampicin mono-resistant;

* - Fisher's Exact test.

**Figure 1:**
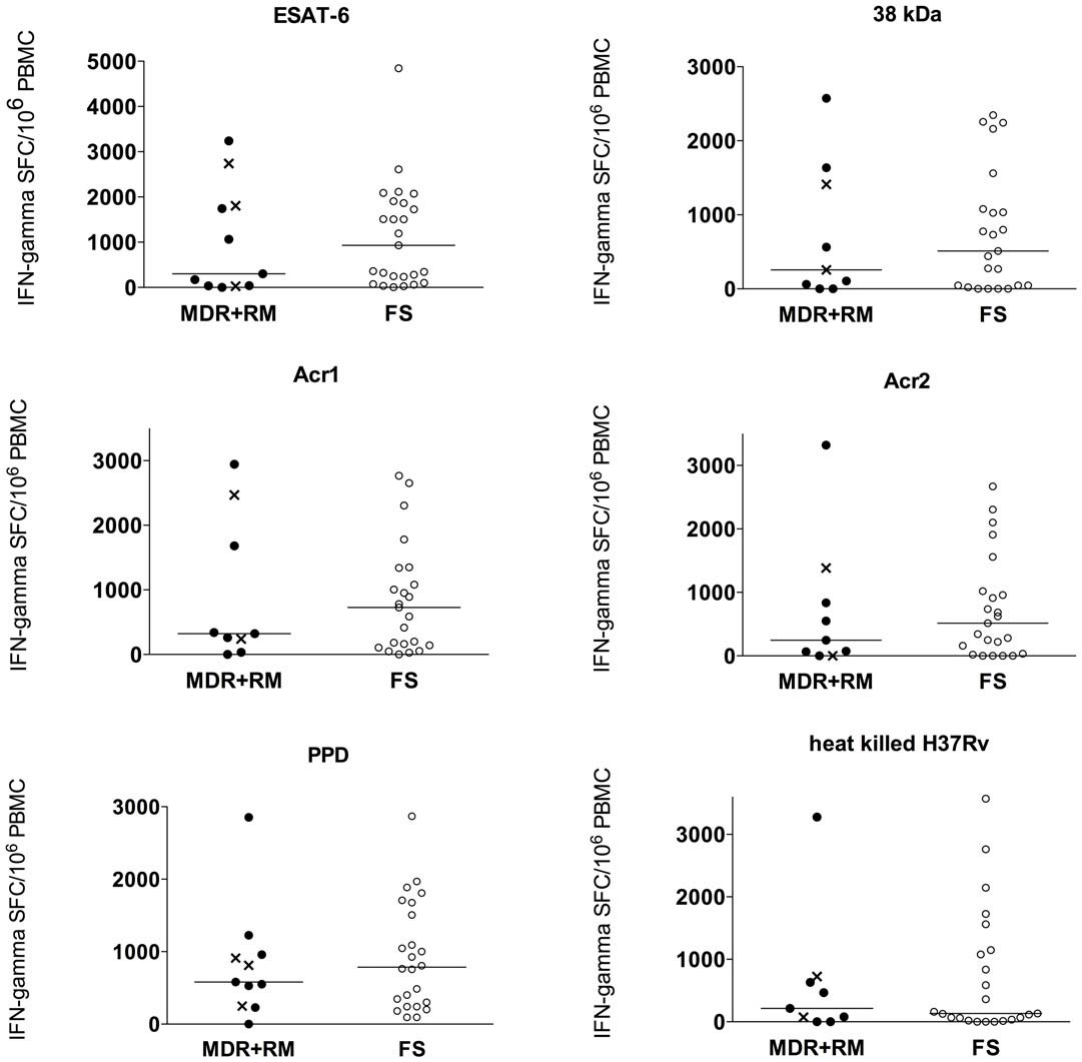
IFN-gamma ELISpot response of peripheral blood mononuclear cells (PBMC) stimulated with a range of *Mycobacterium tuberculosis* antigens, expressed as IFN-gamma spot forming cells per million (SFC/10^6^) PBMC. TB-IRIS patients with multi-drug resistant (MDR) TB are shown in black full circles, rifampicin mono-resistant (RM) cases are shown as a cross, and fully sensitive (FS) TB cases are shown as open circles.

The IFN-gamma ELISpot response to various MTB antigens was not statistically different between the patient groups (Figure 1). The median response to ESAT-6 tended to be higher in the FS group, with 931 (IQR 168-1887) IFN-gamma SFC/10^6^ PBMC, compared to 300 (34-1808) in the MDR+RM group, although the difference was not statistically significant (p = 0.53). The response to the 38 kDa antigen also tended to be higher in the FS group, 510 (47-1078) SFC/10^6^ PBMC compared to 255 (30-1523) in the MDR+RM group (p = 0.9). A similar trend towards higher responses in the FS group to the alpha-crystallins Acr1 (median 727 IQR 141-1340, compared to 322 IQR 137-2076 in the MDR+RM group) and Acr2 (median 516 IQR 33-1018, compared to 247 IQR 34-1111 in the MDR+RM group) was observed (both p>0.5). Responses to PPD and hkH37Rv were not different between the groups: median 582 (IQR 248-958) vs 785 (IQR 256-1633) to PPD, and 214 (IQR 37-678) vs 133 (IQR 33-1146) to hkH37Rv in the MDR+RM and FS groups respectively (p = 0.5 in both cases).

The concentrations of 17 cytokines and chemokines in the supernatant of PBMC cultures with and without stimulation from TB-IRIS patients with drug-sensitive (FS, n = 10) and drug-resistant (MDR, n = 10) disease are shown in Table 2, with comparative p values corrected for multiple comparisons using Bonferroni correction. Immune activation was evident in both groups of patients, as shown by the significant increase in inflammatory cytokine concentrations of TNF, IL-1βeta, and IL-6 (FS group only), upon stimulation of PBMC with hkH37Rv. Similarly, a significant increase in the concentration of IL-10 was detected upon stimulation in both groups, however, this tended to be greater in the MDR group (median 310 pg/ml compared to median 163 pg/ml in the FS group, p>0.5). Of the Th-1 type cytokines, IFN-gamma and IL-2 significantly increased upon stimulation in both groups (to median 759 and 132 pg/ml respectively), but to a lesser extent in the MDR group (to median 103 and 34 pg/ml respectively). Changes in IP-10 (CXCL10) concentrations were not significant in either group following correction for multiple comparisons, however the increase upon stimulation in the FS group was 11.5 fold greater compared to the MDR group. Of the chemokines known to attract Th1 cells via CCR5: MIP-1alpha (CCL3) and MIP-1beta (CCL4) were significantly increased upon stimulation in both groups. Similarly, the neutrophil chemoattractant IL-8 (CXCL8) was significantly increased in both groups, as well as GM-CSF. The Th2 cytokines IL-4, IL-5 and IL-13 were essentially undetectable in these samples. Of note, the baseline characteristics and ELISpot responses of the subset of 10 FS patients included in the cytokine determinations did not differ from the full set of FS patients (data not shown).

**Table 2:** Multiplex cytokine analysis (corrected for multiple comparisons).

Median pg/ml *IQR*	FS (n = 10) unstimulated	FS (n = 10) stimulated	p(1)	MDR (n = 10) unstimulated	MDR (n = 10) stimulated	p(2)	p(3)
TNF	202	40597	0.013	213	18177	0.001	>1
	*190-237*	*12326-80459*		*182-250*	*3212-76079*		
IL-1beta	77	9422	0.006	112	7285	0.026	>1
	*77-152*	*1349-24296*		*93-212*	*191-37595*		
IL-6	545	114700	0.026	2200	107578	0.26	>1
	*229-7752*	*47775-246122*		*114-6020*	*10829-282391*		
IL-10	12	163	0.001	10	310	0.013	>1
	*8-19*	*101-1037*		*6-44*	*126-1090*		
IFN-gamma	2	759	0.001	1	103	0.018	>1
	*1-2*	*63-6906*		*1-1*	*15-841*		
IP-10 (CXCL10)	2958	151311	0.26	1262	13159	>1	>1
	*1067-6454*	*7948-726412*		*606-3303*	*1199-289846*		
IL-12p40	0	33	0.052	0	33	0.86	>1
	*0-3*	*14-350*		*0-2*	*0-130*		
IL-2	1	132	0.003	0	34	0.018	0.89
	*0-1*	*66-1234*		*0-0*	*9-123*		
MIP-1alpha (CCL3)	1428	279656	0.003	1252	40981	0.013	>1
	*1-1887*	*115527-397732*		*278-2069*	*15793-331725*		
MIP-1beta (CCL4)	822	90146	0.008	168	47396	0.013	>1
	*638-4299*	*58661-167796*		*76-1119*	*19502-199932*		
RANTES (CCL5)	23194	82344	>1	18091	55152	>1	>1
	*6574-85501*	*20247-102345*		*4604-52786*	*20975-183281*		
IL-8 (CXCL8)	3200	82256	0.003	7422	78256	0.01	>1
	*233-20728*	*61533-93089*		*0-16700*	*53533-93923*		
GMCSF	0	3901	0.001	96	1676	0.004	>1
	*0-0*	*1227-5655*		*65-106*	*510-7852*		
IL-15	3	3	N/A	0	0	N/A	N/A
	*2-3*	*2-4*		*0-0*	*0-0*		
IL-4	1	2	N/A	0	0	N/A	N/A
	*0-1*	*1-4*		*0-0*	*0-0*		
IL-5	0	1	N/A	0	0	N/A	N/A
	*0-0*	*0-1*		*0-0*	*0-1*		
IL-13	5	8	0.08	4	12	>1	>1
	*3-5*	*5-53*		*4-9*	*5-25*		

(1) p value comparing FS unstimulated with FS stimulated.

(2) p value comparing MDR unstimulated with MDR stimulated.

(3) p value comparing FS stimulated with MDR stimulated.

N/A: comparison not applicable due to very low values.

**Figure 2:**
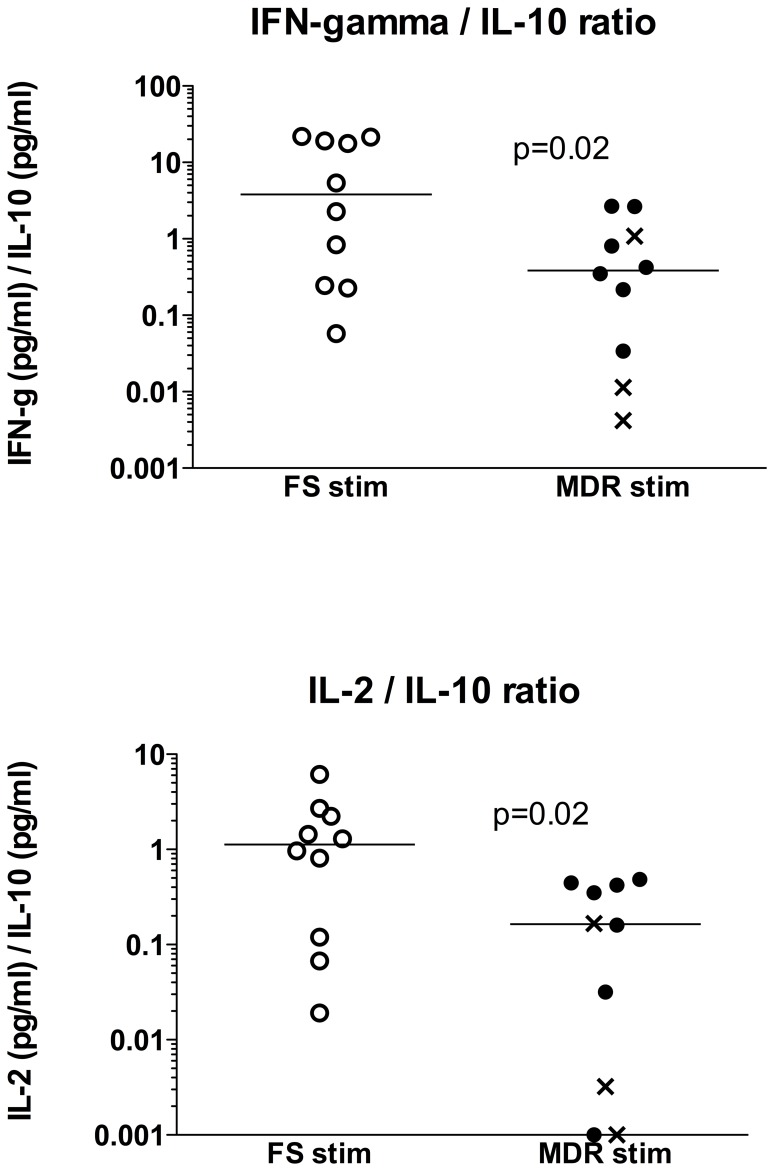
The ratio of secreted cytokines measured by luminex analysis in the supernatants of peripheral blood mononuclear cells (PBMC) stimulated with heat killed H37Rv (MOI = 1∶1) for 24 hours. Results from TB-IRIS patients with multi-drug resistant (MDR) TB are shown in black full circles (n = 7), rifampicin mono-resistant (RM) cases are shown as a cross (n = 3), and fully sensitive (FS) TB cases are shown as open circles (n = 10).

Because of the opposing trends of the Th1 cytokines and the regulatory IL-10 concentrations observed between the MDR and FS groups, we next evaluated the ratio of IFN-gamma/IL-10 in PBMC stimulated with hkH37Rv, and found it to be significantly higher in the FS group (median 3.8 IQR 0.2-19.7) compared to the MDR group (median 0.38 IQR 0.03-1.08), p = 0.02 (Figure 2). A similar trend was observed for the IFN-gamma induced protein CXCL10 (IP-10), with the ratio of IP-10/IL-10 being 305 (IQR 53-1666) in the FS group, compared to 86.6 (IQR 5.3-421) in the MDR group (p = 0.2, data not shown). The ratio of IL-2/IL-10 was also significantly higher in the FS group (median 1.13 IQR 0.1-2.4) compared to the MDR group (median 0.16, IQR 0.0-0.4, p = 0.02, Figure 2). The individual patient's data on hkH37Rv stimulated release of IFN-gamma, IL-2, IL-10, and the respective ratios of IFN-gamma/IL-10 and IL-2/IL-10 are summarised in Table 3. While there was variability between patients in the two groups (Figure 2), the IFN-gamma/IL-10 ratio of 7/10 patients in the FS group was higher than the median ratio seen in the MDR group. In the same time, 10/10 patients in the MDR group had an IFN-gamma/IL-10 ratio lower than the median seen in the FS group. The same was observed for the IFN-gamma/IL-2 ratio as well. In order to further relate cytokine release to the ELISpot responses, we also included the response to ESAT-6 and the heat killed whole bacilli (hkH37Rv) on an individual patient basis in Table 3. We found a significant correlation between the ESAT-6 response and IFN-gamma release into the supernatant of PBMC stimulated with hkH37Rv in the FS group (Spearman r = 0.73, p = 0.02), indicating agreement between the readout of Th1 immunity using two different systems. Since this correlation was only significant in the FS group (the corresponding Spearman r = 0.16, p = 0.65 in the MDR group), it indirectly points towards impaired Th1 immunity in the MDR group that may be related to higher bacterial load. These data overall point towards an altered balance between the Th1 and regulatory cytokine responses in TB-IRIS patients in whom the cause of TB is an MDR strain compared to those with a fully sensitive strain.

**Table 3:** Individual patient's data on hkH37Rv stimulated release of IFN-γ, IL-2, IL-10, ratios of IFN-gamma/IL-10, IL-2/IL-10 and ELISpot responses to ESAT-6 and hkH37Rv.

Patient group	Patient identifier	IFN-gamma pg/ml	IL-2 pg/ml	IL-10 pg/ml	IFN-gamma/IL-10	IL-2/IL-10	ESAT-6 SFC/10^6^ PBMC	hkH37Rv SFC/10^6^ PBMC
**FS**	43	296	99	5149	0.06	0.02	1510	585
	62	10000	2402	1864	5.36	1.29	2610	1725
	72	14571	1096	761	19.15	1.44	2073	360
	77	1203	152	68	17.66	2.24	1866	126
	83	2270	287	106	21.47	2.71	2114	834
	110	39	12	173	0.23	0.07	342	1146
	113	5874	1649	269	21.86	6.14	101	1561
	118	314	112	138	2.27	0.81	931	3571
	215	71	82	84	0.84	0.97	74	0
	257	37	18	152	0.24	0.12	34	67
*Median*		*759*	*132*	*163*	*3.82*	*1.13*	*1221*	*710*
**MDR**	19	43	12	3763	0.01	0.00	1808	
	50	921	122	349	2.64	0.35	3240	465
	52	814	125	752	1.08	0.17	2740	725
	79	1	0	20	0.03	0.00	300	0
	105	3007	544	1124	2.67	0.48	34	630
	127	19	24	53	0.35	0.45	1742	3277
	141	120	63	150	0.80	0.42	0	80
	196	86	33	204	0.42	0.16	174	214
	238	1	0	270	0.00	0.00	27	74
	247	233	34	1079	0.22	0.03	40	0
*Median*		*103*	*34*	*310*	*0.38*	*0.16*	*237*	*214*

## Discussion

We studied the immune response of TB-IRIS patients in whom the cause of TB was a drug resistant strain compared to those with a fully sensitive strain, with the hypothesis that there would be a difference between the groups. We found an altered balance between the Th1 and regulatory responses with decreased Th1 (IFN-gamma and IL-2) and increased IL-10 cytokine secretion in the drug resistant group.

We recently described in a cross-sectional study that TB-IRIS is associated with hypercytokinemia [16]. Here we used the same panel of cytokines to explore the hypothesis that there is a difference between the immune response in TB-IRIS patients whose TB was caused by fully sensitive compared to those with drug-resistant MTB. Our hypothesis was suggested by data indicating that patients with MDR-TB have enhanced regulatory T cells and impaired Th1 responses [15], [18]. We wished to determine whether this finding would stand in the context of TB-IRIS induced hypercytokinemia.

The IFN-gamma ELISpot responses to a range of MTB antigens showed no statistically significant differences between the two groups of patients, although a trend towards higher responses to the RD-1 encoded secreted antigen ESAT-6 in the FS group was noted. Frequencies of circulating ESAT-6 specific IFN-gamma secreting T cells were previously described to be higher in patients with minimal disease and low bacterial burdens compared to patients with culture positive active disease [19]. Since all patients were on first line therapy at the time of diagnosis with paradoxical TB-IRIS and sampling, it is possible that the bacterial load of the patients in the MDR+RM group was higher, due to failed therapy, compared to those with fully sensitive TB. Indeed, the response to the 38 kDa cell wall associated protein and the intracellular Acr1 and Acr2 tended to be higher in the FS group, possibly indicating that treatment in this group results in more bacterial destruction, thus these antigens becoming accessible to circulating, antigen specific T cells in the blood. Having previously shown large expansions of IFN-gamma secreting cells in HIV-TB co-infected patients who develop TB-IRIS as well as in similar controls who do not develop IRIS [17] it was perhaps not surprising that our present results showed no difference between the two groups of TB-IRIS patients.

We also evaluated cytokines secreted into the supernatant of peripheral blood mononuclear cells stimulated overnight with heat killed whole bacilli. We found that stimulation resulted in increased cytokine/chemokine concentrations in both groups with a few exceptions, however without a significant difference between the two groups. A reduced ratio of IFN-gamma/IL-10 has been found to relate to increased disease severity in pulmonary and extrapulmonary TB [20]. Disease progression in MDR-TB patients has also been associated with the detection of IL-10 in whole blood [21]. IL-10 is an anti-inflammatory cytokine secreted by many hematopoietic cells and has a central role in infection by limiting the immune response to pathogens in order to prevent damage to the host [22]. However, by suppressing macrophage and dendritic cell functions required for the capture, control, and initiation of immune responses to MTB, it might also be linked to the mechanisms by which MTB evades the host immune response, mediating long-term infection in the lung [23]–[25]. While we found no significant difference between the two groups of patients in the concentrations of IL-10 secreted by MTB stimulated PBMC, we did find a significant difference in the ratio of Th1 cytokines (IFN-gamma and IL-2) to IL-10, which were significantly lower in the MDR group. Thus, our data is in line with suggestions that protective Th1 responses are impaired in some drug-resistant TB patients. However, host immune responses are dependent on the bacterial load, and as discussed earlier, the bacterial load of the patients in the MDR group may be higher due to failed therapy, we hypothesise that the impaired cytokine balance is likely to be caused by the poorly controlled bacterial growth in these patients.

Limitations of our work include (i) the small sample size, (ii) the lack of mycobacterial strain typing and (iii) the lack of cellular analysis. In a previous study however, we showed that TB-IRIS is not associated with increased proportions of regulatory CD4^+^FoxP3^+^ T cells [17]. Moreover, Guyot-Revol et al have shown that while FoxP3 expression is increased in patients with TB, IL-10 and TGF-β1 mRNA did not correlate with regulatory T-cell markers [26], suggesting that the source of IL-10 in these TB patients could be other cell types. Indeed, it has been shown that the frequency of MTB antigen specific CD8+ T cells secreting IL-10 is elevated in MDR-TB patients [27].

MTB is a successful pathogen because it has the ability to persist in an immune competent host via a number of immune evasion strategies, such as altered antigen presentation to prevent the recognition of infected macrophages by T cells, and evading macrophage killing mechanisms [28]. While some of these mechanisms are more prominent in heavily infected cells, the resulting immune response will be dependent on the bacterial load. Determining the tuberculous bacterial load and its link to pathogenesis remains one of the greatest challenges in our understanding of human tuberculosis.
